# Prediction of cabbage seed drying at lab and industrial scale using a ‘non-equilibrium’ sorption isotherm model

**DOI:** 10.1016/j.crfs.2025.101239

**Published:** 2025-11-04

**Authors:** J. Veser, W. Peeters, M.A.I. Schutyser, R.G.M. van der Sman

**Affiliations:** a Laboratory of Food Process Engineering, Wageningen University and Research, P.O. Box 17, Wageningen, 6700 AA, the Netherlands; b Food Biobased Research, Wageningen University and Research, P.O. Box 17, Wageningen, 6700 AA, the Netherlands

**Keywords:** Cabbage seeds, Seed drying, Priming, Drying model, Isotherm, Stresses

## Abstract

Drying is an important step in seed treatments to ensure high seed quality as well as storability. As drying can also negatively affect seed quality and is a time and energy intensive process, a mechanistic drying model was built to improve trial-and-error research and apply it to fluidised bed trials. First, material characteristics such as the sorption isotherm and diffusivity were measured and modelled. For the isotherms, it was found that a ‘non-equilibrium’ isotherm described the drying data better than the fully equilibrated measured isotherm. For the ‘non-equilibrium’ isotherm, the Flory-Huggins model was extended with an addition in glassy state including an elastic parameter by Leibler & Sekimoto. The different sorption behaviour during drying is hypothesized to occur due to accumulated mechanical stresses in the material. Microscopy and X-ray tomography support this hypothesis as air pockets in the seed appear during drying. The single seed model including the ‘non-equilibrium’ isotherm could predict drying behaviour of thin-layer dried seeds at different drying conditions up to 40 °C very well with a percent error below 10 %. Further, lab- and industrial-scale fluidised bed trials were performed, which were very reproducible and comparable. The uniformity of mixing in fluidised beds was checked to assess the applicability of the single seed model. Mixing was uniform and the single seed model could predict fluidised bed drying well. Thus, the model has high predictive power for moisture decrease for thin-layer as well as fluidised bed drying at lab and industrial scale. Limitations are drying temperatures above 40 °C, due to a not-optimal diffusivity model and no isotherm data. The proposed model can be a useful tool for optimising seed drying processes, if combined with a quality model as a next step.

## Highlights

• Mechanistic model can predict drying curve of thin-layer dried cabbage seeds.
• Flory-Huggins model with elastic parameter correctly described drying isotherm.
• Microscopy/XRT show proof of mechanical stress on material during drying.
• Mixing in fluidised bed was uniform, so drying model can be used for fluid bed.
• The model can predict moisture decrease for fluid bed at lab and industrial scale.

## Graphical abstract

**Figure:**
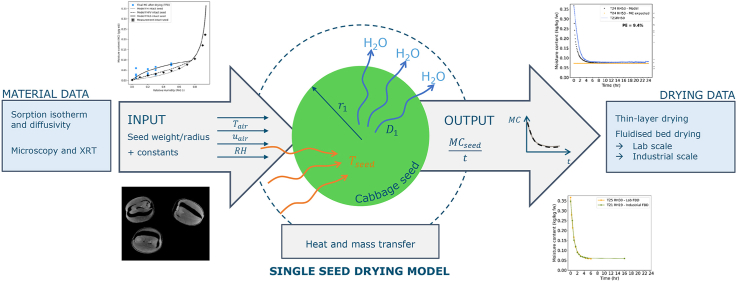

## 1 Introduction

Drying is an important step in vegetable seed treatments such as priming. Priming enhances seed germination speed and uniformity on the field and consists of pre-hydrating and drying of seeds (Bewley et al., 2013; Sano et al., 2017). Drying is done to ensure high quality as well as storability after pre-hydrating the seeds. However, severe drying can damage the seeds and decrease seed quality, i.e. germination capacity, up to viability loss of the embryo (Veser et al., 2025). Therefore, drying conditions are critical for seed quality management. Current seed drying protocols are based on empirical knowledge. They are usually conservative with low temperatures and long drying times and therefore suffer from low energy efficiency (Jittanit, 2007). To improve the performance of drying operations, better understanding of the drying kinetics of seeds is key to develop more knowledge-based guidelines. In a first experimental study, the drying kinetics of cabbage seeds and the effect on seed quality were extensively characterised (Veser et al., 2025). The current study extends our work by model development describing cabbage seed drying kinetics in detail.

In literature the drying kinetics of seeds are often described by empirical thin-layer drying models such as the Page, Weibull and Newton model (Jittanit, 2007; Benestante et al., 2024; Borges-Machado et al., 2024; Doymaz, 2016; Doymaz and Pala, 2003; Ismail et al., 2019; Bissaro et al., 2023; Raimundini Aranha et al., 2024; Malekjani et al., 2013; Roberts et al., 2008). These models use an effective diffusivity in the order of 10^−10^ to 10^−11^ m^2^ s^−1^ and an Arrhenius-type relation to describe the temperature dependency of the drying kinetics with an activation energy of ∼30 kJ mol^−1^. Only few studies employ a more physics-based approach by numerically solving Fick's law for various types of seeds and even fewer consider coupled heat and mass transfer or even shrinkage (Bruce, 1985; Johann et al., 2016; Watanabe et al., 2023; Haibo and Zhao, 2012). Moreover, Baidhe and Clementson (2024) highlighted in their review about modelling of grain and seed drying that only few grain types are investigated and thus the applicability of models and recommendation for other crops is limited. Thus, more species-specific research is needed.

The objective of this study is the development of a numerical model describing the drying behaviour of a vegetable seed for sowing, which can later be used for developing knowledge-based guidelines for process control, when including a seed quality model in the future. Further, we want to investigate whether a single seed model still applies in fluidised beds, and whether the fluidised bed is well mixed.

Our numerical model considers coupled heat and mass transfer and shrinkage of a single seed during drying. Material properties of the seed like diffusion coefficient and sorption isotherm are determined via independent experiments. Both thin-layer and fluidised bed drying experiments were carried out for model validation. As in our previous paper, *Brassica oleracea* (cabbage) seeds were selected in this study being a relevant vegetable seed for sowing and having a spherical shape facilitating simpler model development. Brassica crops (such as cabbages, broccoli, cauliflower and others) are important horticultural products, being the 3rd most important vegetable in the world as determined by weight in 2022 (FAO; Veser et al., 2025). Cabbage seeds are spherical seeds with a diameter of ca. 2 mm and consist of mainly embryo material including the first root (radicle) and first leaves (cotyledons, oil-rich), very little endosperm and a seed coat surrounding the embryo and endosperm (Appelqvist, 1971).

The seed drying model builds on the model developed earlier for predicting drying of single droplets of food materials (Siemons et al., 2022). Thin-layer drying experiments were performed using the method developed in our previous study (Veser et al., 2024). Further drying experiments were conducted using fluidised bed dryers at both the laboratory and industrial scales. Additional mixing tests were performed to assess the uniformity of the laboratory fluidised bed system.

## 2 Model description

The numerical seed drying model was based on a previously developed single droplet model as described in more details below (Siemons et al., 2022). It is based on heat and mass transfer equations and iteratively solves at what rate water diffuses from the inside to the outside of a seed and evaporates at the surface. It is assumed that the seed is spherical in shape and is heating up uniformly, with no temperature gradient within the seed. Key components of the drying model are an accurate sorption isotherm model, which predicts the final moisture content at a certain drying condition, and a valid diffusivity model for the seed material.

### 2.1 Sorption isotherm modelling

The sorption isotherm of seeds was described by the Flory-Huggins free-volume theory as shown in Eq. (1) (Fox and Flory, 1950; Flory, 1953; van der Sman and Meinders, 2011). The effective interaction parameter χ_eff_ (−) is composition dependent following Eq. (2), which interpolates between the interaction parameter in the concentrated regime χ_ws_ and that of the semi-dilute regime χ_0_ = 0.5 (van der Sman and Meinders, 2011). For the concentrated regime parameter of the solid seed material, the equation was fitted to the measured desorption data above glass transition. The moisture content in the glassy state was determined with the Couchman-Karasz model as described in a previous study (Veser et al., 2024). Fitting was done with the curve_fit function in Python, which is based on the least squares method.

(1) $$a_{w}=\exp\left(\frac{\mu_{w}}{R_{gas}\;T}\right)=\exp\left(\ln\left(\varphi_{w}\right)+\left(1-\frac{1}{N_{s}}\right)\cdot \left(1-\varphi_{w}\right)+\chi_{eff}\cdot {\left(1-\varphi_{w}\right)}^{2}+\mu_{FV}\right)$$

(2) $$\chi_{eff}=\left\{ \begin{array}{c} \chi_{ws},\chi_{ws}\leq \chi_{0}\\ \chi_{ws}-\left(\chi_{ws\;}-\chi_{0}\right)\cdot {\left(\varphi_{w}\right)}^{2},\chi_{ws}>\chi_{0} \end{array}\right.$$

Variables are water activity $a_{w}$ (−), chemical potential $\mu_{w}$ (J·mol^−1^), ideal gas constant $R_{gas}$ (J·kg^−1^·K^−1^), seed temperature *T* (K), volume fraction of water $\varphi_{w}$ (−), the ratio of molar volume of solvent and solute $N_{s}$ (−) and the chemical potential addition $\mu_{FV}$ following the free volume theory.

The free-volume theory for glassy state was added, Eq. (3), (Vrentas and Vrentas, 1991). This addition accounts for distinct behaviour of the sorption isotherm in the glassy state and at different temperatures, approximating more fundamental theories describing viscoelastic behaviour (van der Sman, 2023). Free volume stands for the void space between polymers, in which a penetrant such as water can move. Diffusivity of water and thus the sorption of water is dependent on this free volume according to Vrentas & Vrentas (Fox and Flory, 1950). If there is more free volume due to structural changes of the polymer, e.g. at higher temperature or in glassy state, more water can penetrate and thus be absorbed.

(3) $$\mu_{FV}=\frac{M_{w}{dC}_{p.s}}{R_{gas}T}\;\cdot {\left(1-y_{w}\right)}^{2}\cdot \left(1-\frac{T}{T_{g}}\right)\cdot \frac{\partial T_{g}}{\partial y_{w}}$$

Instead of free-volume theory, we also tested an alternative theory reported by Leibler & Sekimoto (Leibler and Sekimoto, 1993), Eq. (4), who directly couples the extra contribution to $\mu_{w}$ to mechanical stresses occurring during drying if the seed enters the glassy state. The Leibler-Sekimoto theory ($\mu_{lS})$ takes elastic modulus $K_{g}$, the molar volume of water $\nu_{w}$ (m^3^·mol^−1^) and the volume fraction of water at glass transition temperature $\varphi_{w,Tg}$ (−) as parameters:

(4) $$\mu_{FV}\to \;\mu_{LS}=\frac{\nu_{w}}{R_{gas}T}\cdot \;K_{g}\cdot \log\left(\frac{1-\varphi_{w}}{1-\varphi_{w,Tg}}\right)$$

Cabbage seeds have a high amount of oil content of ca. 30 % based on dry weight (Veser et al., 2024). Oil, however, does not contribute to the water absorption. Therefore, the volume fraction of water was calculated only from the mass fraction of water $y_{w}$ and the mass fractions of hygroscopic solids $y_{i}$ with the density of water $\rho_{w}$ and solid $\rho_{i}$ via Eq. (5):

(5) $$\varphi_{w,o}=\frac{\frac{y_{w}}{\rho_{w}}}{\sum_{i}\frac{y_{i}}{\rho_{i}}+\frac{y_{w}}{\rho_{w}}}$$

The densities of hydroscopic solids including sugars, complex carbohydrates, protein, fibres (= also complex carbohydrates, same density) and ash were calculated based on composition and temperature according to Phinney et al. (2017). The composition of primed cabbage seeds was determined as described in a previous study and can be found in the Appendix (Table A1) (Veser et al., 2024).

### 2.2 Diffusivity modelling

Diffusivity of water in seed material $D_{w}$ (m·s^−2^) was described with an empirical logarithm function of mass fraction $y_{w}$ (−) and an Arrhenius term for including the temperature dependency with activation energy *E*_*a*_ (J·mol^−1^), Eq. (6), (Räderer et al., 2002).

(6) $$D_{w}=\left(a\cdot \log_{10}\left(y_{w}\right)+b\right)\;\cdot \exp\left(\frac{-E_{a}}{R_{gas}}\;\cdot \;\left(\frac{1}{T}-\frac{1}{298.15\;K}\right)\right)$$

Parameters *a* and *b* were fitted to measured diffusivity data of whole cabbage seeds at our reference temperature 25 °C = 298.15 K with the curve_fit function in Python. The activation energy *E*_*a*_ was fitted with the same method to our experimental data of the diffusion coefficient at 40 °C.

### 2.3 Numerical seed drying model

The seed drying model was based on numerical models previously developed to describe single droplet drying of polymer solutions (Siemons et al., 2022; Adhikari et al., 2007; Mezhericher et al., 2010; Perdana et al., 2013; van der Sman, 2003). The temperature during drying is assumed uniform within the seed as the Biot number, Bi < 0.4 (Patel and Chen, 2008). All assumptions made for this model were (a) a uniform temperature of the seed, (b) a perfect sphere geometry, (c) one homogenous material and (d) ideal shrinkage.

Heat and mass transfer were calculated based on differential equations. We solved the equations in the co-moving Lagrangian reference frame of the shrinking seed with the convective time/material derivative $D_{t}=\partial_{t}+\nabla \cdot \vec{u_{s}}$. This led to equations in the form of Fick's, Eq. (7), and Fourier's law. As we can assume uniform temperature, Fourier's law could be integrated over the volume, and the volume integral could then be transformed into a surface integral via the Gauss theorem (Whittaker, 1935). The boundary condition was implemented, which led to Eq. (8) for describing the heat transfer:

(7) $$D_{t}c_{w}=\nabla \cdot D_{m}{\nabla c}_{w}$$

(8) $$\rho_{eff}c_{p,eff}V\;\frac{dT}{dt}=\left(h_{eff}\left(T–T_{air}\right)\;–\left[\;\Delta H_{evap,0}+\left(c_{p,v}–c_{p,w}\right)\left(T–T_{0}\right)\;\right]\;J_{evap}\right)A$$

Variables are the water mass concentration $c_{w}$ (kg·m^−3^), the velocity of the moving solids $u_{s}$ (m·s^−1^), the mutual moisture diffusion $D_{m}$ (m^2^·s^−1^), the effective density $\rho_{eff}$ (kg·m^−3^), the effective heat capacity $c_{p,eff}$ (J·kg^− 1^ K^− 1^), the seed volume *V* (m^3^), the drying time t (s), the seed temperature T (K), the effective heat transfer coefficient $h_{eff}$ (W·m^−2^·K^−1^), the temperature of the bulk air $T_{air}$ (K), the heat of vaporization of water at 0 °C $\Delta H_{evap,0}$ (J·kg^−1^), the specific heat of vapour $c_{p,v}$ and water $c_{p,w}$ (J·kg^− 1^ K^− 1^), the reference temperature for the enthalpy for evaporation $T_{0}$, being 0 °C = 273.15 K, the evaporative flux $J_{evap}$ (kg·m^−2^·s^−1^) and the surface area of the seed A (m^2^).

Initial and boundary conditions were that (A) the seed temperature and moisture content were both uniform at the start, (B) that there was no influx of water in the middle of the seed and (C) that the diffusive flux equals the evaporative flux at the surface of the seed and that (D) heat transfer via the surface comes from the temperature difference of seed and air and the needed energy for evaporation:

(9) $$A:{c_{w}}_{\left(t=0\right)}=c_{w,0}\;T_{\left(t=0\right)}=T_{0}$$

(10) $$B:-D_{m}\partial_{r}{c_{w}}_{\left(r=0\right)}=0$$

(11) $$C:{-D_{m}\partial_{r}c_{w}}_{\left(r=R\right)}=J_{evap}$$

(12) $$D:{-\lambda_{eff}\partial_{r}T}_{\left(r=R\right)}=h_{eff}\left(T–T_{air}\right)\;–\left[\;\Delta H_{evap,0}+\left(c_{p,v}–c_{p,w}\right)\left(T–T_{0}\right)\;\right]\;J_{evap}$$

Variables are the radial coordinate r (m), the seed radius R (m) and the initial water concentration $c_{w,0}$ (kg·m^−3^). The evaporative flux was described as follows:

(13) $$J_{evap}=\frac{\beta_{ext}\;M_{w}}{R_{gas}T_{avg}}\left(a_{w}p_{sat}\left(T\right)-{RH}_{air}p_{sat}\left(T\right)\right)\text{,}$$

with the external mass transfer coefficient $\beta_{ext}$ (m·s^−1^), the water activity $a_{w}$ (−) following Eqs. (1)–(4), the partial vapour pressure in air $p_{air}={RH}_{air}p_{sat}\left(T\right)$, the relative humidity of air ${RH}_{air}$ (−) and saturation pressure $p_{sat}$ (Pa) at seed temperature T (K) and the estimated average temperature of the boundary layer $T_{avg}=\left(T_{air}+2T\right)/3$ (K).

**Fig. 1:**
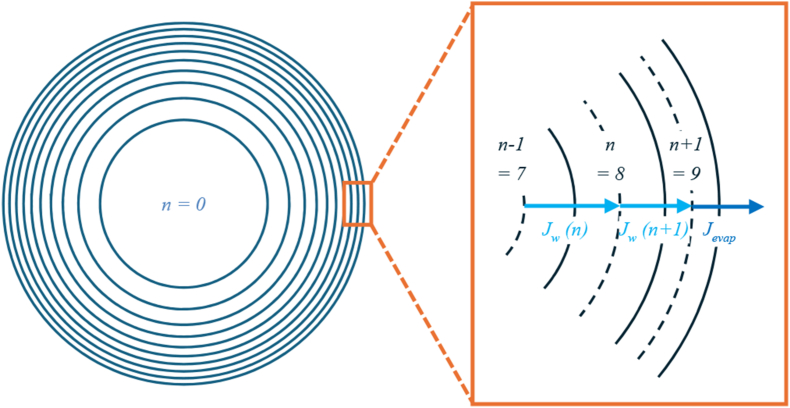
Sketch of equal volume elements (shells, n) of Finite Volume Method and visualisation of diffusive (J_w_) and evaporative flux (J_evap_).

We solved the mass balance in spherical coordinates applying the cell-centred Finite Volume Method by dividing the seed into *N* = 10 shells of equal volume (Fig. 1), including central differencing and simple Euler forward time integration. Decreasing the number of shells to *N* = 5 led to an increased percent error by 0.1 %, increasing the number of shells up to *N* = 20 led to the same percent error as with *N* = 10. *N* = 10 was chosen as computing time was faster. The Lagrangian frame of the solids was used as the solid material is assumed incompressible and thus remains constant in each control volume. Only water is exchanged between control volumes, leading to volume change of the spherical shells. For each time step, new volumes, radii, surface areas and average concentrations of the control volumes are computed. Based on these values, the new diffusive mass and energy fluxes are calculated.

The concentration at the centre of each control volume is used for the calculations (Fig. 1). For calculating the evaporative flux at the surface, the centred concentration of the outer shell was found to be a good approximation for the surface moisture concentration. The equations for $\beta_{ext}$ and $h_{eff}$ can be found in Table 1. Values of model parameter, material specific parameter and input parameter can be found in Table A2, A3 and A4.

**Table 1:** Closure equations used in drying model. The values of the parameters are indicated in Table A2 and for material specific parameters the values can be found in Table A3.

Equation name	Equation	Reference
External mass transfer coefficient	$\beta_{ext}=\frac{Sh\;D_{air}}{2R}$	Siemons et al. (2022)
Sherwood number (Ranz Marshall)	$Sh=2+0.6{Re\;}^{0.5}{Sc}^{0.33}=2+0.6{\left(\frac{U2R}{\nu_{air}}\right)}^{0.5}{\left(\frac{\nu_{air}}{D_{air}}\right)}^{0.33}$ With the speed of air U = 0.235 m s^−1^ and the radius of the seed R (m)	Ranz and Marshall (1952), Adhikari et al. (2007)
Heat transfer coefficients	$h_{eff}=\frac{1}{\frac{1}{h_{\mathrm{int}}}+\frac{1}{h_{ext}}}$ with $h_{ext}=\frac{Nu\;\lambda_{air}}{2R}$ and $h_{\mathrm{int}}=\frac{\lambda_{eff}}{0.25\;R}$	van der Sman (2003)
Thermal conductivity	$\lambda_{eff}=\varphi_{w}\;\lambda_{w}+\varphi_{s}\;\lambda_{s}$	Siemons et al. (2022)
Nusselt number Ranz Marshall	$Nu=2+0.6{Re\;}^{0.5}\;{\mathrm{Pr}}^{0.33}=2+0.6{\left(\frac{U2R}{\nu_{air}}\right)}^{0.5}{\left(\frac{\nu_{air}}{\alpha_{air}}\right)}^{0.33}$ with $\alpha_{air}$ as thermal diffusivity of air (m^2^·s^−1^)	Adhikari et al. (2007)

One variation of the model was tested adapting assumption (c): one homogenous material. Instead of a single, uniform material, the seed was assumed to consist of two different materials, i.e. the coat and embryo material. In that case, the most outer shell *N*-1 = 9 represented the seed coat and the other shells the embryo material. This means that the sorption isotherm model, i.e. the fitted interaction parameter, was different for these two materials, otherwise there would be no difference in materials. Due to different isotherm behaviour of the materials, the flux from shell 8 (embryo) to 9 (coat) was implemented based on water activity difference instead of concentration difference according to Eq. (14) adapted from Chen et al. (2017):

(14) $$J_{w,\left(N-1\right)}=\frac{\beta_{\mathrm{int}}\;A_{\left(N-2\right)}c_{w,avg}}{a_{w,avg}}\left(a_{w,\left(N-2\right)}-a_{w,\left(N-1\right)}\right)\text{,}$$

with internal mass transfer coefficient $\beta_{\mathrm{int}}$, the area of the second most outer shell $A_{\left(N-2\right)}$ (m^2^), the average water concentration $c_{w,avg}$ and the average water activity $a_{w,avg}$ (−) of the second most outer (*N*-2) and most outer shell (*N-*1).

For all other shells 0 to 8, the diffusion flux based on water concentration difference was kept. For the evaporation flux, the water activity of the coat material was used.

## 3 Materials and methods

### 3.1 Materials

Packaged cabbage seeds (Brassica oleracea) were supplied by Bejo Zaden B.V., The Netherlands. The dry and untreated seeds were stored frozen at −20 °C for long-term storage and at 4 °C for mid-term storage to slow deterioration during storage. Frozen seed can survive much longer than non-frozen. Short-term storage was performed at RH = 30 % and T = 15 °C. Seed packages were always hermetically closed and before opening seeds were equilibrated to room temperature.

### 3.2 Sorption isotherm

Sorption isotherms were determined with a vapour sorption analyser SPS (ProUmid GmbH & CO. KG, Germany). The equilibrium condition was set to 0.001 % weight change per 60 min. The minimum weighing cycle time was 60 min and the maximum time was 48 h, the time between the weighing cycle was 10 min. First, the samples were kept at 25 °C and 0 % RH for 20 h to ensure dry seeds. From there, the following humidity steps were measured: 0, 20, 40, 60, 80, 95 % RH. For the desorption curve, more steps were taken: 95, 80, 70, 60, 50, 40, 30, 20, 10, 0 % RH. Whole seeds as well as only embryo or only seed coat material were measured, ca. 100 mg each, and in duplicate. Hydrated seeds were gently cut open with a scalpel and then the coat and embryo material were separated with tweezers.

### 3.3 Diffusivity

Diffusivities were measured using a dynamic vapour sorption analyser Discovery SA (TA Instruments, USA). Three seeds were placed in a pan, which was tared and weighted for 24 h at constant conditions of 25 °C and 30 %. Air inflow was fixed at 200 ml min^−1^. Whole seeds as well as only embryo and coat material were analysed.

From these drying curves, the diffusivity of water in the material was calculated following the regular regime method as described and verified by Schoeber (1976), Perdana et al. (2014) and Siemons et al. (2019). The regular regime method assumes that the system is in a steady state when the moisture gradients are not changing. Thus, dimensionless Fickian diffusion equations can be solved to obtain the concentration-averaged diffusivity through a slab with a certain thickness (Schoeber, 1976; Perdana et al., 2014). Two conditions need to be verified for use of the regular regime method. The initial water content as well as the drying air should not be limiting to mass transfer and influence the result. Both conditions were verified and are discussed in section 4.2.

### 3.4 Drying with lab-scale fluidised bed

Before drying, the seeds were hydrated for 4 days on a roller mixer in 100 mL tubes with a 1 mm hole, each tube had 3 g of seeds. It was 160 % weight-based water added based on the dry matter content of the starting material. After that, seeds had a moisture content (MC) of ca. 36.7 %, less than theoretically achievable by the hydration most likely due to evaporation of water during mixing of 4 days. Moisture content was determined with the oven method at 105 °C for 16 h. After hydration, seeds were directly subjected to drying.

**Fig. 2:**
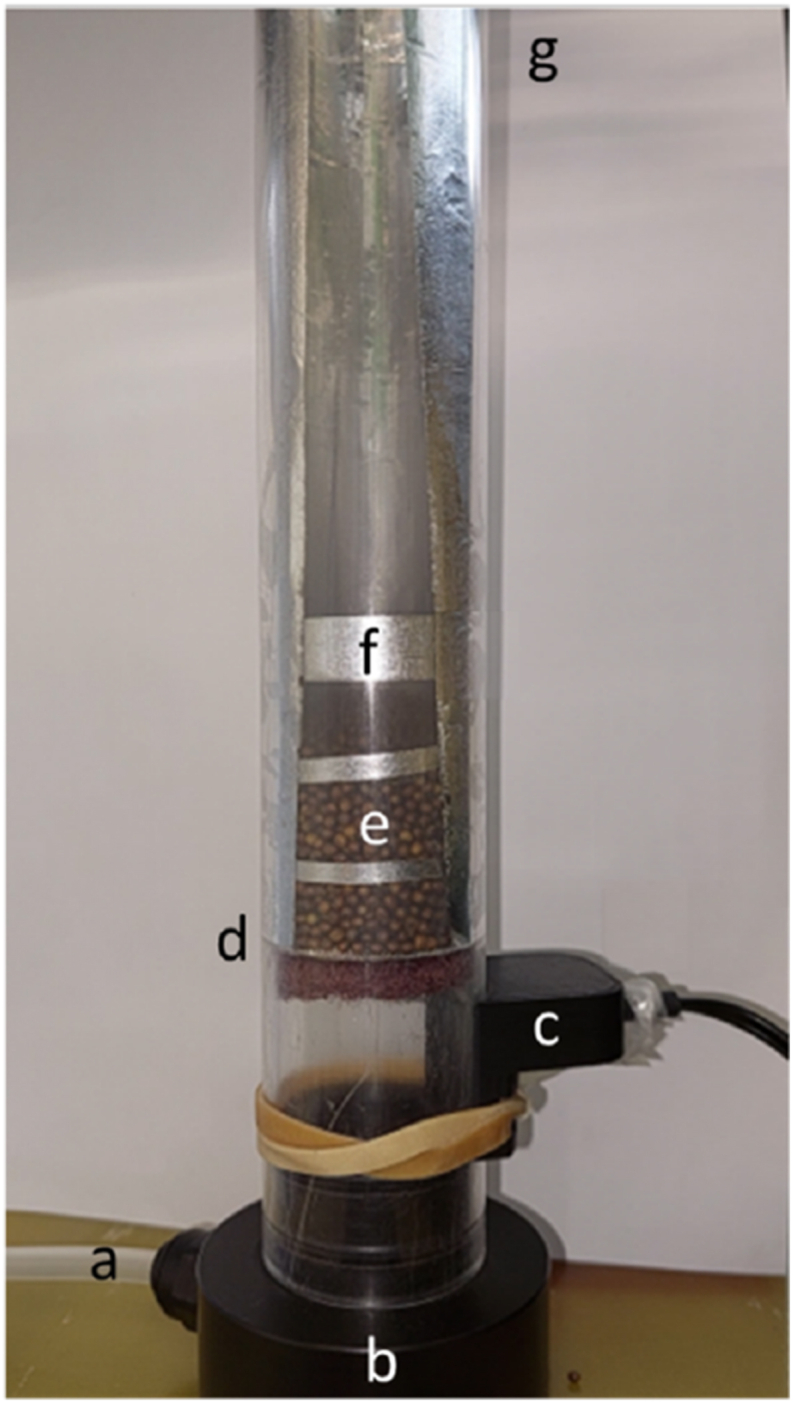
Lab-scale fluidised bed column with a diameter of 32 mm. Air inlet (a), standing on scale (b), temperature and relative humidity sensor air inlet (c), exchangeable mesh (d), seed bed (e), aluminium foil to prevent electrostatics (f), air outlet and temperature and relative humidity sensor air outlet (g).

A custom-built lab-scale fluidised bed dryer was constructed consisting of a 32 mm diameter acrylic tube with a fine 0.5-mm mesh included (Fig. 2). The air inlet was positioned close to the bottom. Two sensors were inserted on the side close to the air inlet and at the outlet measuring RH and T of ingoing and outgoing air. A highly porous lid was put on top of the column to avoid possible spilling of seeds but allowing air to pass. An exchangeable mesh was placed close to the bottom of the column to support the seed bed and facilitate air distribution. The dryer was connected to a humidifier with a CEM-unit (Bronkhorst, The Netherlands), which can control the velocity, temperature and RH of the ingoing drying air. Further, the fluidised bed dryer column was placed on a mass balance. The humidifier was programmed to stop every hour for weight measurement. Therefore, the air cable needed to be manually detached. Batches of 15 g of seeds were dried at a condition of 25 °C and 30 % RH with two different air speeds 60 and 85 ln·min^−1^. Minimum fluidisation speed was calculated to be ca. 55 ln·min^−1^. Drying times varied from 30 min to 2 h. Trials were performed in duplicate. Drying conditions will be abbreviated in the following as T25RH30, e.g., indicating the value of the drying air temperature (T) and air relative humidity (RH).

### 3.5 Image analysis to assess seed mixing behaviour

Seeds were coloured with spray paint in black and white and images were taken from the front view of the seed bed every 2 min. Seed were painted only for specific fluidised bed experiments that evaluate mixing behaviour, otherwise seeds were not painted. Each image was analysed by a custom-made image analysis script in Python. First, each image was turned into a black and white image and the pixels were divided into black and white pixels. The picture was divided into 25 imaginary squares and for each square the number of black and white pixels and consequently the fraction of white pixel could be calculated. Based on this fraction, the mixing entropy was calculated for each image, thus time step. The mixing entropy was calculated following the procedure and equations reported by Koerten et al. (van Koerten et al., 2015).

### 3.6 Drying in an industrial-scale fluidised bed

Before drying at the industrial scale, seeds were hydrated for 4 days in a rolling drum with a hole in the lid and 160 % water added. Batches consisted of 1.5 kg of seeds in a drum of 20 L.

**Fig. 3:**
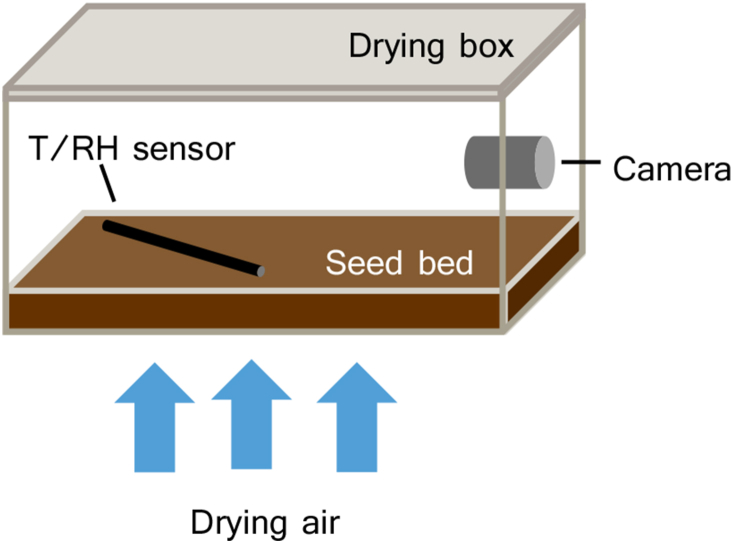
Schematic set-up of industrial fluidised bed dryer.

After hydration, 1.5 kg of seeds were transferred into a rectangular box of 41.8 L and 0.0988 m^2^ with a mesh below (mesh size of 0.415 mm) (Fig. 3). These boxes were placed in a drying wall, where from the bottom conditioned drying air was blown through the seed boxes (Rijk Zwaan B.V., The Netherlands). The air flow varied between 550 and 3200 m^3^ h^−1^ according to a ramping up protocol to achieve fluidisation. The drying air was controlled on temperature and humidity (via the moisture content of the air) by a heater and a desiccant air dryer. Part of the drying air was recirculated and mixed with fresh air, either ambient air or dried ambient air to reach the required moisture content. After that, the drying air was heated to the set temperature. Inlet and outlet air conditions were monitored with sensors at the inlet and outlet. Seed bed temperature and RH were measured with a Aranet T/RH IP67 sensor lying in the seed bed. Manually, every hour seed samples were drawn and the MC was determined with a moisture analyser HB43 (Mettler-Toledo GmbH, Switzerland). Three different drying conditions were tested in duplicate (T25RH30, T25RH20, T40RH30).

### 3.7 Microscopy

Thin layers of seeds were prepared for light microscopy. Therefore, seeds were submerged in a square cavity with pure OCT (optimal cutting temperature) mounting medium (VWR Int B.V.), a colourless water soluble blend of glycols, that served as a specimen matrix for cryo-sectioning of the seeds. Subsequently, a vacuum pump was applied to remove any air bubbles and the samples were frozen to - 30 °C. The frozen blocks were mounted on a sample holder in a cryo-microtome to make thin seed slices (∼20–50 μm) at ca. −18 °C. These slices were transferred by directly melting them onto object slides (at RT) and were mounted in OCT with a coverslip. The samples were analysed with the light microscope Eclipse 80i (Nikon Europe B.V.) with 4x (Plan Fluor NA 0.13) and 10x (Plan Fluor NA 0.5) objectives equipped with a DSFi3 Nikon camera and with the stereo microscope Discovery V12 (Carl Zeiss AG, Germany) at a magnification of 35.2x equipped with an Axicam MRc5.

### 3.8 X-ray tomography (XRT)

For 3D non-invasively and non-destructively imaging, a GE Phoenix v|tome|x m tomographer (General Electric, Wunstorf, Germany) was used. The seeds were placed in a plastic straw which was folded and placed in the sample holder. The folded straw kept the seeds in place and minimized moisture loss. The system contains two X-ray sources. The 240 kV micro focus tube with tungsten target was used. X-rays were produced with a voltage of 80 kV and a current of 60 μA.

The images are recorded by a GE Dynamic41|200 detector array with 2024 × 2024 pixels (pixel size 200 μm). The detector is located 815 mm from the X-ray source. The object was placed 19.6 mm from the X-ray source. This results in a spatial resolution of 5.0 μm. A full scan consists of 1500 projections over 360°. The 1st image is skipped. The saved projection is the average of 3 images, where every image is obtained over 250 ms exposure time. Only for selected samples, an extended measurement time (30 averages as compared to 3) was used to obtain a better signal to noise ratio.

GE reconstruction software version 2.10.1 - RTM (Wunstorf, Germany) was used to calculate the 3D structure via back projection. The 3D images, obtained using the v|tome|x XRT, are analysed using Avizo imaging software version 2021.2.

### 3.9 Statistics and model accuracy

Statistics and model equations were implemented in Python 3.12. using packages numpy, pandas, matplotlib, seaborn, scipy, openpyxl and math. Measurements are shown as mean ± standard deviation or confidence interval of 95 %.

Model accuracy is evaluated via percent error PE (%), Eq. (15), (Roberts et al., 2008; Mclaughlin and Magee, 1998; Lomauro et al., 1985). A PE of smaller than 10 % is generally considered as a high accuracy. n represents the number of data points used for comparison, ${MC}_{\exp}$ and ${MC}_{model}$ are the moisture content values at the same time obtained by experiment and by numerical modelling respectively.

(15) $$\text{PE}=\frac{100}{n}\;\sum_{i=1}^{n}\frac{\left|{MC}_{\exp,i}-{MC}_{model,i}\right|}{{MC}_{model,i}}$$

### 3.10 Use of artificial intelligence

10.13039/100004318Microsoft Copilot was used as a support during programming with Python for the drying model. It was used to generate initial code as a starting point according to our ideas, mainly for the creation of graphs from the data, or to suggest improvements for existing code when errors occurred. We always reviewed and adapted the code snippets to ensure correctness and relevance for our research.

## 4 Results and discussion

### 4.1 Sorption isotherm model for cabbage seeds

**Fig. 4:**
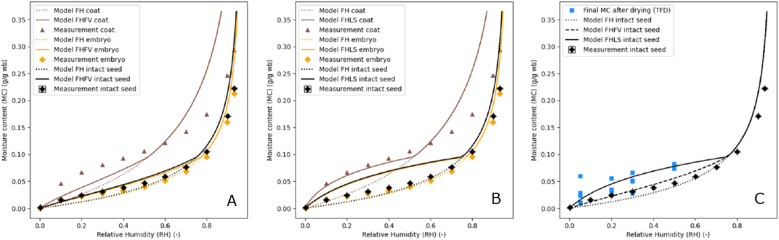
A. Sorption isotherms measured at 25 °C for whole cabbage (circle with error bars, black), embryo (triangle, yellow) and coat material (triangle, brown) and modelled with Flory-Huggins (FH) model and an adaption at glass transition according to Free-Volume theory (FV), with chi1 = 1.244 for whole cabbage, chi1 = 1.302 for embryo and chi1 = 0.465 for coat material. B. Sorption isotherms measured at 25 °C for whole cabbage (circle with error bars, black), embryo (triangle, yellow) and coat material (triangle, brown) and modelled with Flory-Huggins (FH) model and an adaption at glass transition according to Leibler and Sekimoto (1993) (LS). C. Sorption isotherms measured at 25 °C for whole cabbage (circle with error bars, black) and final moisture content data of drying experiments (TFD thin film dryer) (square, blue), and Flory-Huggins model with adaptions FV and LS in glassy state.

Sorption isotherms were determined for whole cabbage seeds, embryo material and coat material. In Fig. 4A, only the desorption curve is shown as this is the relevant material behaviour during drying. In our previous study, the full isotherm of cabbage seeds is shown (Veser et al., 2024). We observed a small hysteresis between the sorption and desorption curve which occurred after the glass transition. Fitting the Flory-Huggins theory to the experimental data resulted in an interaction parameter χ_ws_ = 1.244 for whole cabbage, χ_ws_ = 1.302 for the embryo material and χ_ws_ = 0.465 for the coat material. Whereas whole cabbage and embryo material showed the same behaviour, the coat material showed a distinct different isotherm. This different water desorption behaviour might indicate a different composition of coat and embryo material. A major difference is most probably that the coat has only 15 % oil content in contrast to the 35 % oil that we have measured for the whole seeds (Table A1) (Appelqvist, 1971). Further, the material has a higher water-holding capacity upon drying than the embryo material. It may be that the seed coat has a higher fibre content, which have a higher water-holding capacity. The standard Flory-Huggins (FH) model describes the sorption isotherms for the whole and embryo material very well, but not that of the coat material (Fig. 4A). FH strictly only holds above the glass transition (van der Sman and Meinders, 2011). Therefore, for moisture contents in the glassy state an extra term is added to calculate water activity based on the free volume theory by Vrentas & Vrentas (Vrentas and Vrentas, 1991) (see Eq. (3)). This addition improved the goodness of fit clearly. The percent error between isotherm model and experimental data reduced for all three materials, for the coat from 49 % to 41 %, for the embryo from 32 % to 13 % and for the whole cabbage from 33 % to 8 %. The values are obtained excluding the value at 0 % RH as this had a too big influence on the error calculation, skewing the error evaluation. Especially for lower temperatures, the materials equilibrate to a higher moisture content in glass transition than as expected due to an elastic contribution, which arises due to “slow structural relaxation in the glassy state” and which leads to more water retention (van der Sman et al., 2013).

In addition, we compared the final moisture contents of seeds after the drying trials, assuming they have reached equilibrium with the drying air and thus should in principle match with the isotherm of whole seeds (Fig. 4C). However, we observed that the seeds during drying retain more water if compared to the measured sorption isotherm. This may have two reasons: 1) Seeds may have not yet reached equilibrium yet as the drying was shorter (1 day) then the isotherm measurement (ca. 2 weeks), 2) Because the drying rate was faster during the drying and not step-wise like in the isotherm measurement, the sorption equilibrium could have shifted to higher moisture contents. Most likely mechanical stresses that arise during drying cannot relax and thus affect the material behaviour. When the seeds experience more mechanical stresses, i.e. elasticity, it may lead to increased water retention (van der Sman et al., 2013; Hu et al., 2023). This reasoning leads to the conclusion, that for successful prediction of shorter and faster seed drying a ‘non-equilibrium’ isotherm or modelling of mechanical stresses is required. It depends on the structure of the model if the ‘non-equilibrium’ isotherms of the seed coat and seed embryo or the whole seed is needed. For a model assuming one material, the isotherm of whole seeds is used. For a model assuming two materials, the isotherm of the seed coat and seed embryo should be used.

**Fig. 5:**
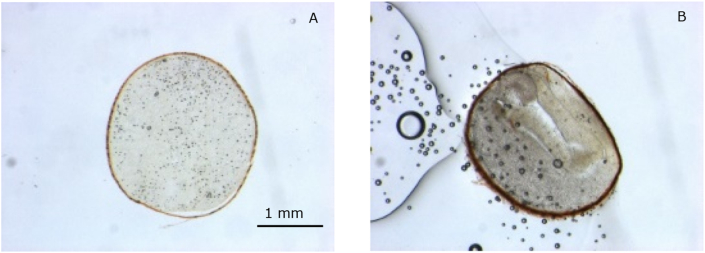
Light microscopy of cabbage, thin slice prepared with cryo-tomography. A. Hydrated cabbage B. 24 hr dried cabbage at 25 °C and 30 % RH.

**Fig. 6:**
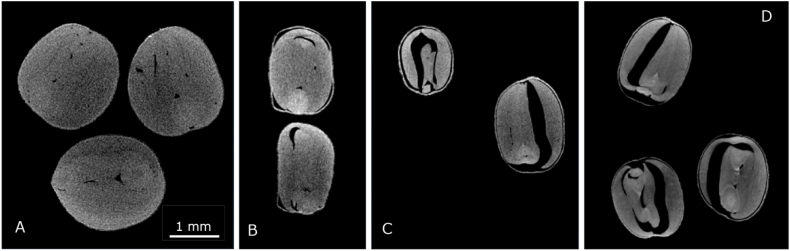
XRT images of cabbage seeds, 2D projections extracted from 3D images. Different drying times starting with hydrated seeds and 0 h (A), 0.5 h (B), 3 h (C) to 24 h (D) of drying at 25 °C and 30 % RH.

We analysed dried seeds with light microscopy and XRT to further understand the possible mechanical stresses and its influence on the seed tissue (Fig. 5, Fig. 6). As seen with microscopy and XRT, big air pores have built up between the embryo layers during drying, which was not expected. However, this phenomenon of pore formation, which is still quite unknown to the drying community, was previously observed in maltodextrin droplets (Siemons et al., 2020) and further explained and modelled by van der Sman (van der Sman et al., 2024a, 2024b). Pore formation can take place during drying of a viscoelastic material, when a hard, elastic shell or skin arises that goes into glassy state. This happens to the seed coat. When drying continues, stresses cannot relax away during the time scale of seed drying as due to the glassy state viscoelastic relaxation times become very large. Thus, the shell does not further shrink due to developed stresses. Also, within the material core stresses develop. The material core shrinks, also to compensate the volume loss of continuous water evaporation. Stresses at the surface of small pre-existing pores within the material result in a gas pressure drop and thus an expansion of these pores. Thus, the observed pores in the XRT images proof that mechanical stresses arise in cabbages seeds during drying. These stresses also lead to a different water holding capacity of the material which indicates the necessity to include mechanical modelling, such as an elastic parameter, in the isotherms (van der Sman, 2023; van der Sman et al., 2013). Further, we think the pores won't affect drying per se as diffusivity of water through air is much faster than through the material (Appendix, Table A2). Thus, diffusion through the material is still governing the process. However, if the pore becomes very large, the water diffusion trajectory might decrease, which can enhance the drying. Especially, if the pore is connected to the outside of the seed, drying can become faster because the diffusion trajectory is reduced, and drier air can penetrate the pore. Although, larger pores have been observed to be formed during drying, differences in mass transfer are expected small. This is confirmed in section 4.3, where the drying model could predict drying very well without considering air pores. Moreover, measured diffusivity probably effectively captured the effects of pores, because that may also have happened during the independent diffusivity experiments.

Leibler & Sekimoto (Leibler and Sekimoto, 1993) proposed an isotherm model with an elastic parameter, the bulk modulus *K*_g_ (Pa). If this term is added for the glassy state, the isotherm model appeared to better predict the final MC of the dried seeds from our previous study (Veser et al., 2025). Further, this elastic addition provided more accurate prediction of the coat isotherm behaviour indicating that even during slow isotherm measurements stresses were build up in the coat. *K*_g_ was reported to be 1.1∙10^9^ Pa for the polymer PVS or maltodextrin (dextrose equivalent 5) (Siemons et al., 2022; Leibler and Sekimoto, 1993). In this case, the best fit to the final drying moisture contents was found for *K*_g_ = 3.3∙10^9^ Pa, which is in the same order of magnitude. A next step would involve detailed measurements and modelling of the mechanical stresses and their influence on sorption behaviour. Possible measurements for the correct bulk modulus could include dynamic mechanical analysis (DMA) as well as atomic force microscopy (AFM). Seifert et al. (2021) showed that AFM could be used to investigate the viscoelastic properties and relaxation times of seedling tissue and cell walls in vivo.

The formation of pores due to mechanical stresses could be included in the model, which may further improve the prediction of the final MC and can give insight into the right elastic modulus. More data and research are needed for that. For our purpose, the elastic modulus in the isotherm by Leibler & Sekimoto was good enough and is a good first approach to describe the abnormality of the isotherm due to elasticity.

### 4.2 Diffusivity model for cabbage seeds

**Fig. 7:**
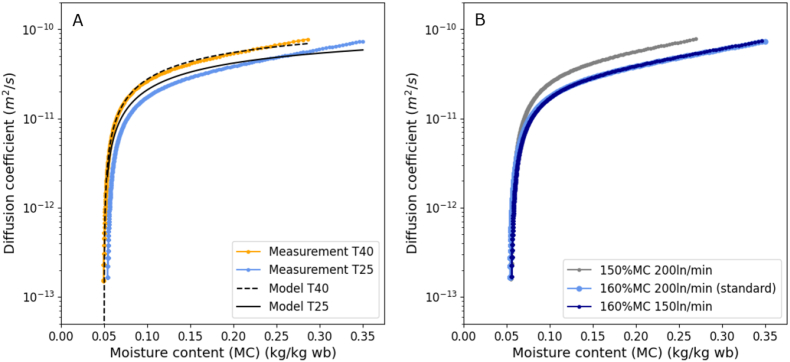
A. Diffusivity data at 25 °C (blue) and 40 °C (orange) air temperature (T) was obtained with a dynamic vapour sorption device and fitted a logarithmic model (black). B. Diffusivity data at 25 °C (blue) with different starting moisture contents (MC, wet based (wb)) (added water based on the dry weight, after hydration of 4 days real MC ca. 37 %) and different air speeds in ln/min of the drying air.

The measured diffusion coefficient of whole cabbage seeds slightly decreased with decreasing MC and below ca. 10 % MC it rapidly approached zero (Fig. 7). A logarithmic model was chosen to describe diffusivity as function of moisture content. In addition, using an Arrhenius term the temperature dependency of the diffusivities was described (Räderer et al., 2002). Parameters *a* and *b* were fitted to measured diffusivity data of whole cabbage seeds at 25 °C and were found to be *a* = ${3\cdot 10}^{-11}$ and *b* = ${9\cdot 10}^{-11}$ with the curve_fit function in Python. The activation energy *E*_*a*_ was fitted with the same method to our experimental data of the diffusion coefficient at 40 °C and found to be 14.18∙10^3^ J mol^−1^. As described by van der Sman (van der Sman, 2023), the diffusivity most likely levels off instead of reaching zero in the glassy state and at lower MC, thus, we applied a lower limit for the diffusivity at 1.0∙10^−12^ m s^−2^.

The method of measuring diffusivity is prone to error. Three whole spherical seeds were measured and from the obtained drying curves the diffusivity varying with moisture content was extracted using the regular regime method (Perdana et al., 2014; Siemons et al., 2019). This regular regime method is based on three assumptions: (1) a thin layer of the material with same thickness, geometry like a slab; (2) the drying is independent of the initial moisture content and (3) the drying is independent of speed of drying air. Assumption (2) and (3) were checked (Fig. 7B). Varying air flow did not influence the results and varying initial water content only slightly influenced the measured diffusivities. We concluded that assumption (3) is met and assumption (2) is not really met, but still in the similar regime. Also, assumption (1) is more difficult to meet as we did not want to mill the seeds and used whole seeds, which are not like a layer of material. This is not ideal, but acceptable as a first approximation. For the regular regime method, we used the seed radius as the height of the material slab through which water needs to diffuse. Overall, this method resulted in reasonable diffusion coefficient values comparable to values in the order of 10^−10^ to 10^−11^ m^2^ s^−1^ observed in previous studies (Jittanit, 2007; Benestante et al., 2024; Borges-Machado et al., 2024; Doymaz, 2016; Doymaz and Pala, 2003; Ismail et al., 2019; Bissaro et al., 2023; Raimundini Aranha et al., 2024; Malekjani et al., 2013; Roberts et al., 2008).

### 4.3 Drying model for cabbage seeds

#### 4.3.1 Thin-layer drying

**Fig. 8:**
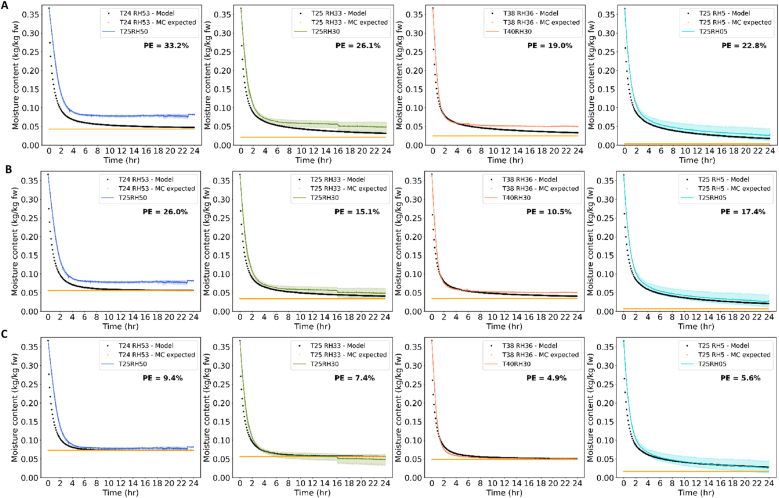
Four different drying conditions with experimental data from thin-layer drying in colour and the predicted data in black (one-material model), in orange final MC predicted based on the isotherm model. Percent error (PE) between model and data is shown. A. Flory-Huggins isotherm model B. Flory-Huggins and free-volume theory isotherm model C. Flory-Huggins and Leibler-Sekimoto addition isotherm model. Drying conditions indicated with inlet air temperature (T) in °C and relative humidity (RH) in %.

The obtained sorption isotherm of whole cabbage seeds (Fig. 4C) and diffusivity equation were implemented into the numerical heat and mass transfer model for predicting drying of a single spherical seed. The numerical model predictions were first compared to the drying curves obtained from thin-layer experiments reported by Veser et al. (2024) (Fig. 8). The results are presented as mean with a 95 % confidence interval of several trials per drying conditions. For the drying conditions 25 °C and 50 % or 30 % RH, a step in the mean can be seen, which is due to different drying times of the trials ranging from 1.5 h to 24 h. Further, we chose in this study to use the average values of inlet and outlet air temperature and RH as in the previous study. Reason is that these are the more realistic values that the seeds over the seedbed were exposed to. The air temperature and RH differences in our system are also relatively small (e.g. set 40 °C vs average 38 °C) and a different choice would lead to a deviation of maximally 4 % PE.

The model predictions were greatly influenced by the isotherm as already expected and discussed in section 4.1. For the isotherm including the elastic parameter, the model described thin-layer drying for four different drying conditions very well, the percent error was similar to or below 10 % (Fig. 8C). This percentage is usually accepted as good description (Roberts et al., 2008; Mclaughlin and Magee, 1998). The isotherm with FH model or FHFV model describes the data much worse with errors ranging from 16 to 35 % (Fig. 8A and B).

**Fig. A1:**
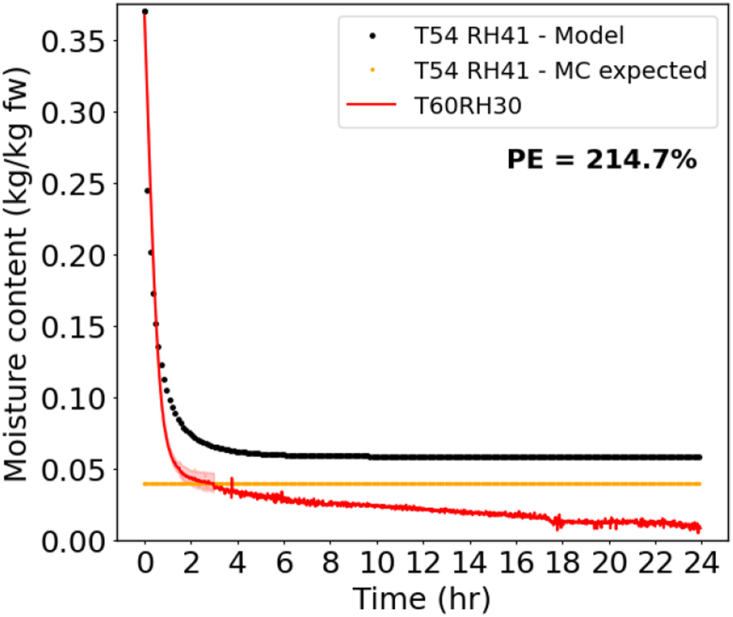
Drying curve at 60 °C with experimental data from thin-layer drying in red and the predicted data in black (one-material model), in orange final MC predicted based on the isotherm model: Flory-Huggins and Leibler-Sekimoto addition isotherm model. Drying conditions indicated with inlet air temperature (T) in °C and relative humidity (RH) in %. The model does not predict experimental data correctly (high percent error (PE)), demonstrating the limit of the current model.

The model is limited in validity at temperatures above 40 °C as we have seen with drying data at 60 °C (Appendix, Figure A1). This limit is most likely due to inaccuracies in calculated diffusivity and sorption values, which again can be explained by the absence of independent measurements at these conditions.

The less accurate predictions for higher temperatures emphasise the importance for establishing complete sorption isotherm and diffusivity relationships for an accurate drying model. It is more challenging to develop accurate models for heterogenous biological systems such as seeds than for more homogeneous systems such as maltodextrin. Nevertheless, this relative simplified mechanistic single seed model assuming only one material and considering mean isotherm and diffusivity characteristics could well predict the drying behaviour of the seeds within the region of process conditions that are most relevant.

**Fig. 9:**
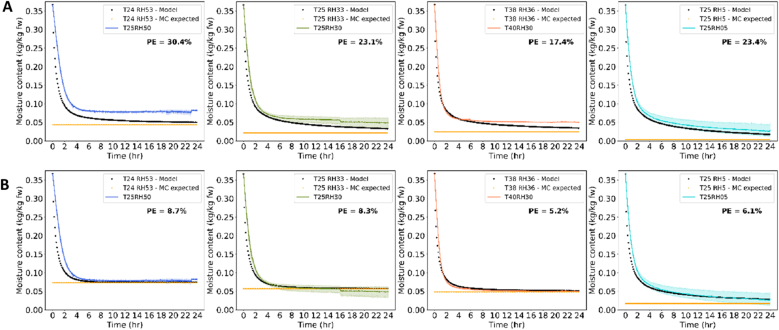
Four different drying conditions with experimental data from thin-layer drying in colour and the predicted data in black (two-material model), in orange final MC predicted based on the isotherm model. Percent error (PE) between model and data is shown. A. Flory-Huggins isotherm model B. Flory-Huggins and Leibler-Sekimoto addition isotherm model. Drying conditions indicated with inlet air temperature (T) in °C and relative humidity (RH) in %.

A final modification of the model was tested to distinguish between the two major tissues in the seed, i.e. the embryo and the coat (Appelqvist, 1971). The most outer shell in the numerical model was chose to represent the coat material and the other shells the embryo material regarding the isotherm model. This approach provided similar predictions as for the previous model, for two conditions slightly better, for two slightly worse (Fig. 9). Looking at the sorption isotherms, the whole cabbage or whole embryo isotherm with elastic contribution describes the final MC very well, whereas the coat isotherm is slightly above the ‘non-equilibrium’ isotherm. Thus, including the coat isotherm will result in a slightly, but not relevant different prediction. In principle, we may use both the single-material and the two-material model, but if we have a correct isotherm model, we also may use the simple one-material model for accurate prediction.

The proposed model is a quite simple mechanistic model, however, more complex than the empirical drying models such as the Weibull or Newton model. These are often used in seed drying studies as they are easier for practical applications (Baidhe and Clementson, 2024). The advantage of a mechanistic model is that more insights can be obtained from the model than just the drying curve. It can also predict moisture profiles inside the seed and it can be easily extended to other predictions such as the pore size or the seed quality based on MC, temperature and shrinkage. Moreover, new knowledge can be obtained such as that mechanical stresses build up during drying, which we would have not observed by fitting an empirical model. Finally, the mechanistic model is presumably easy to adapt for other crops as only material specific properties, namely the parameters for the isotherm and diffusivity model need to be adjusted. It is a big challenge in seed drying research as mentioned by Baidhe & Clementson (Baidhe and Clementson, 2024) and Pedrosa et al. (2024) that most often studies cannot be applied to other seed crops due to species-specific behaviour and many studies are only performed with a few popular seed species, such as grains, soybean, bell pepper or *Solanum* (Baidhe and Clementson, 2024; Pedrosa et al., 2024). Therefore, a mechanistic model can serve as a basis for more general applicability. Further research is recommended to proof the applicability of the proposed model to other seed species.

#### 4.3.2 Fluidised bed drying of seeds

**Fig. 10:**
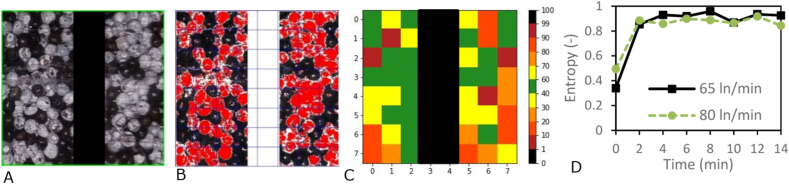
A. Original picture of seeds in the lab fluidised bed B. Modified picture of seeds in the lab fluidised bed (black pixels = black, white pixels = red) C. Heat map of percentage of red pixels in each square D. Entropy calculated based on fractions of white seeds.

Next, we have investigated if the single seed model can be applied to describe fluidised bed drying of seeds, as it is done in practice. We therefore performed fluidised bed drying experiments. A single seed model can be used if we can assume that each seed experiences similar and uniform air conditions in the fluidised bed. Here we assume that the fluidised bed is operated with an air velocity above that of the minimum fluidisation velocity and is thus well mixed. We demonstrated the correctness of this assumption by studying fluidisation of white and black coloured seeds in a lab-scale set-up. It was observed that after seconds the seeds were already well mixed, which was reflected in the entropy of mixing increasing quickly to a maximum value, indicting a well-mixed system (Fig. 10) (van Koerten et al., 2015).

**Fig. 11:**
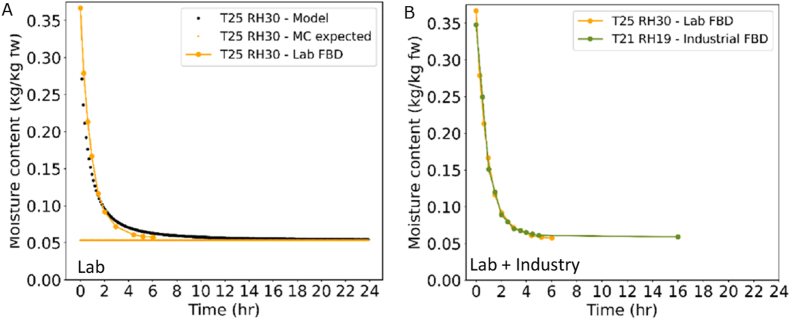
A. Comparison of lab fluidised bed drying (yellow) with predicted data (black). B. Comparison of lab fluidised bed (yellow) with industrial fluidised bed data (green). Drying conditions indicated with inlet air temperature (T) in °C and relative humidity (RH) in %.

Drying curves were also measured in the lab-scale fluidised bed, for which we could also precisely control the drying conditions and measure the weight over time. The obtained drying curves were compared to the predicted drying curves (Fig. 11A). This comparison showed that the model could very well predict fluidised bed drying trials with a percent error below 10 %. This confirms that the assumption of uniform drying conditions is correct. It also indicates that the drying process is limited by mass transfer inside the seeds, which is similar for each individual seed as air conditions are uniform.

Finally, we tested the model to predict drying curves obtained from pilot-scale fluidised bed dryers with batches of 1.5 kg. In these dryers, temperature as well as humidity could be controlled to a certain extent, but not as precisely and not as constant as in the lab-scale set-up. Still, the drying curves were very well comparable to those of the lab-scale fluidised bed (Fig. 11B). For both, lab and industrial scale, only one trial of the duplicates are shown. The reproducibility was very high, which would lead to a very small confidence interval being hardly visible in the graph. It is remarkable that two very different systems, with a different scale, different operators and with different level of precision could produce such similar drying curves. This shows that lab-scale studies can provide relevant data for model development that can be used to optimise also larger-scale processes.

**Fig. 12:**
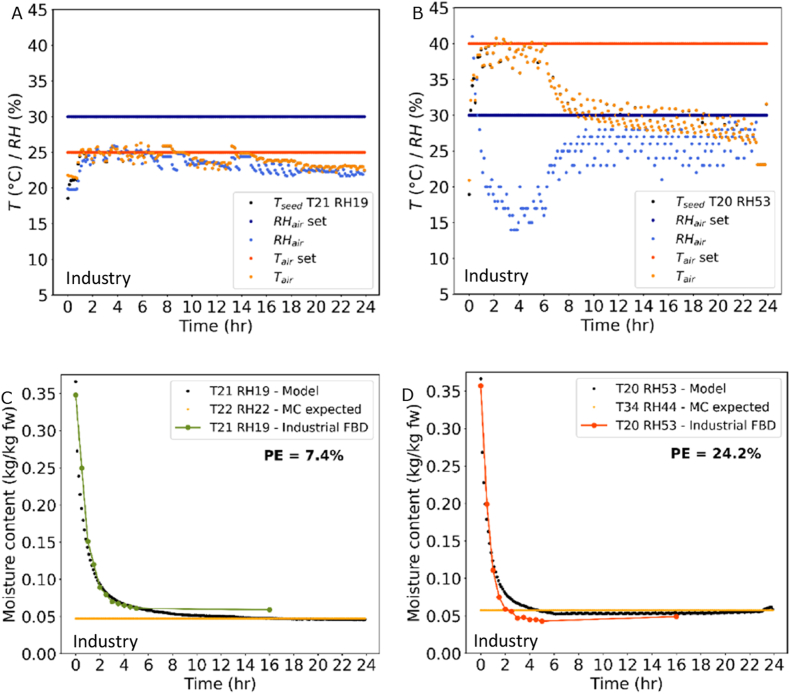
A + B. Input drying air conditions of industrial fluid bed dryer (temperature (T): set - orange, measured - yellow, relative humidity (RH): set – darkblue, measured – lightblue) and measured seed bed temperature (black) on the left. C + D. Drying curves of industrial fluidised bed trial and model (black) with final predicted moisture content (MC) in orange. Percent error (PE) between model and data is shown.

Industrial-scale trials did show varying inlet conditions. We adapted the model so that the measured air conditions were used as input for air temperature and RH (Fig. 12A and B). As shown in Fig. 12C and D, the model could also very well predict the drying kinetics of the pilot-scale fluidised bed dryer. At 40 °C, the initial drying kinetics are captured not so well as for the thin-layer drying trials as seen in Fig. 8C. This deviation might be due to deviations in the input measurements or due to different mixing properties in the industrial dryer, leading to faster drying kinetics. In the initial drying phase, when still more water needs to be evaporated, apparently the drying can still speed up and thus diffusion might be not the limiting factor yet and external drying conditions, such as temperature, RH of the air and well-mixing, can influence drying. Most importantly, the final MC of fluidised bed drying at 40 °C is predicted correctly by the single seed model.

More often, single-sphere models are used for bulk properties of drying processes as they even better predict properties than models with more complex shapes as found for soybean drying by Boac et al. (Baidhe and Clementson, 2024; Boac et al., 2009). Mostly, packed-bed or computational fluid dynamic (CFD) models combined with the discrete element method (DEM) are applied to model the fluidised bed drying process, modelling multiple seeds and the air flow (Baidhe and Clementson, 2024; Li et al., 2023). This adds more insights into the movement of the seeds and air and also more complexity to the model, which we think is not needed for cabbage seed drying. Our experiments have shown that the drying process is mainly governed by internal diffusion if the seeds and air are well mixed, which could be well predicted with a single seed model.

Overall, our hypothesis that single seed model can describe fluidised bed drying correctly was proven. This makes the proposed mechanistic and still relatively simple model a useful tool for both research and practice purposes.

## 5 Conclusions

In conclusion, this study developed a mechanistic single seed drying model with independent determined diffusivity and sorption isotherm models for seed drying. We have found that it could predict drying in thin-layer experiments as well as fluidised bed drying at both lab and industrial scale.

It was found that the use of a ‘non-equilibrium’ isotherm model is crucial for the correct prediction of the final MC. Traditionally sorption isotherms are determined over long times scales, 2–4 weeks, allowing material to obtain equilibrium, meaning that viscoelastic stresses are allowed to relax away. However, drying operations used in practice, as the sowing seed industry, are performed in much shorter time scales (<24h) where viscoelastic stress does not relax away and builds up if the material gets into the glassy state. Thus, the ‘non-equilibrium’ isotherm model can be constructed using Flory-Huggins with an elastic contribution in the glassy state, as done in the Flory-Huggins Free Volume theory or the Leibler-Sekimoto theory. These occurrence of mechanical stresses in drying seeds were evident by the air pore formation in the centre of the seed as observed by microscopy and XRT imaging. With the ‘non-equilibrium’ isotherm model and a simple independently fitted diffusion model, the single seed model could very well predict thin-layer as well as fluidised bed drying at the lab and also at the industrial scale. Limits of the model are temperature above 40 °C and for industrial trials deviations in the initial drying kinetics for 40 °C were seen. Remarkable is the good reproducibility of the lab fluidised bed trials with industrial trials indicating that internal diffusion is mostly the limiting factor of drying, thus the material characteristics are relevant for the drying behaviour and thus also for a successful prediction of seed drying. The proposed model can be a useful tool for optimising seed drying processes, if combined with a quality model as a next step.

## CRediT authorship statement

**Julia Veser:** Conceptualization, Methodology, Formal analysis, Investigation, Writing – original draft. **Ward Peeters:** Methodology, Investigation. **Maarten A.I. Schutyser:** Conceptualization, Methodology, Supervision, Writing – review & editing, Funding acquisition. **Ruud van der Sman:** Conceptualization, Methodology, Supervision, Writing – review & editing.

## Funding sources

This work was supported by the Rijk Zwaan Breeding B.V. and Bejo Zaden B.V.

## Declaration of competing interest

The authors declare that they have no known competing financial interests or personal relationships that could have appeared to influence the work reported in this paper.

## Figures and Tables

**Table A1:** Composition of primed (hydrated and dried) and reference cabbage seeds, dry based (db), measured as reported by Veser et al. (2024).

Component	Reference seeds (g/g db)	Primed seeds (g/g db)
Sugar	0.070	0.068
Carbohydrates (excl. sugars)	0.012	0.042
Proteins	0.359	0.360
Lipids	0.357	0.353
Total dietary fibre	0.155	0.130
Ash	0.046	0.048

**Table A2:** Parameter definitions, values and respective model symbols (adapted from Siemons et al. (2022)).

Parameter	Definition	Value
$c_{p,s}$	Heat capacity of solute (J·kg^− 1^ K^− 1^)	1000
$c_{p,w}$	Heat capacity of vapour (J·kg^− 1^ K^− 1^)	1996
$c_{p,w}$	Heat capacity of water (J·kg^− 1^ K^− 1^)	4200
$D_{air}$	Diffusion coefficient of air (m^2^·s^−1^)	2.42·10^−7^
$\Delta c_{p,s}$	Change in specific heat of solute (J·mol^− 1^ K^− 1^)	420
$\Delta c_{p,w}$	Change in specific heat of water (J·mol^− 1^ K^− 1^)	1910
${dH}_{evap,0}$	Heat of vaporization of water at 0 °C (J·kg^− 1^)	2.50·10^6^
dt	Iteration time step (s)	1
$k_{B}$	Boltzmann constant (J·K^−1^)	1.38·10^23^
N	Total number of shells (−)	10
$p_{0}$	Atmospheric pressure (Pa)	1.00·10^5^
$R_{gas}$	Ideal gas constant (J·K^−1^·mol^−1^)	8.31
$T_{g,w}$	Glass transition temperature of water (K)	139∗
$\eta_{w}$	Viscosity of water (Pas)	1.00·10^−3^
$\lambda_{s}$	Thermal conductivity of solute (W·m^−1^·K^−1^)	0.33
$\chi_{0}$	Interaction parameter of water (−)	0.50

∗In literature, *T*_g,w_ is found to be 139 or 136 K. In the previous study *T*_g,w_ of 139 K was used. Thus, we continue to use 139 K and verified that with both values the same results were obtained, thus it has no further relevant effect on the results.

**Table A3:** Material specific parameter for Brassica oleracea seeds

Parameter	Brassica oleracea seeds	Reference
Molecular weight of maltodextrin *as approximation for solid seed material* without oil M (g·mol^−1^)	474	Siemons et al. (Siemons et al., 2022)
Glass transition temperature of solute $T_{g,s}$ (K)	400	Veser et al. (Veser et al., 2024)
Interaction parameter $\chi_{ws}$ (−)		Fitted to isotherm data
Whole cabbage	1.244	
Embryo material	1.302	
Coat material	0.465	
Elastic parameter $K_{g}$ (Pa)	3.3·10^9^	Fitted to final moisture contents, adapted from Leibler & Sekimoto (Leibler and Sekimoto, 1993)
Activation energy *E*_*a*_ (J·mol^−1^)	14.18·10^3^	Fitted to diffusivity data

**Table A4:** Input parameters for single seed drying model, parameter average values of measurements during thin-layer drying trials.

Parameter	Value
Initial water concentration on wet basis $y_{w}$ (−)	0.367
Oil content on dry basis $y_{o,db}$ (−)	0.3525
Initial seed radius r (m)	1.044·10^−3^
Initial seed temperature $T_{init}$ (°C)	19.3
Air temperature $T_{air}$ (°C)	25–40
Air velocity $U_{air}$ (m·s^−1^)	0.235
Relative humidity of air *RH* (%)	5–50
